# From the Patient's Point of View

**Published:** 1913-06-07

**Authors:** 


### THE EDITOR'S LETTER-BOX.

### From the Patient's Point of View.

*To the Editor of* THE HOSPITAL.

SIR,—Will you allow me space to add a few points by way of explanation to my article which you criticise so leniently in this week's issue of THE HOSPITAL?

1. With regard to the drink of medicine. This, of course, was an aperient, but I resented its being offered to me because I had carefully explained to the authorities already that I had myself taken more than sufficient medicine for this purpose. It was on this ground that I was allowed to enter the Home a day later than would otherwise have been the case.

2. The injection, as I state in my article, was an injection of colouring matter—a species of indigo, I believe—and *not* a narcotic alkaloid. Had the latter been injected as well I would not have complained.

3. Other patients would readily testify to the total inadequacy of the ward utensils.

I trust that my intrusion into your hospitable columns may result in the authorities responsible for this particular Home taking serious notice of my criticisms, which I am prepared to substantiate, if necessary, in the interests of the public at large.—I am, Sir, yours faithfully,

THE EX-PATIENT.

May 30, 1913.

